# Glucagon-like Peptide 1 Receptor Activation Inhibits Microglial Pyroptosis via Promoting Mitophagy to Alleviate Depression-like Behaviors in Diabetic Mice

**DOI:** 10.3390/nu15010038

**Published:** 2022-12-21

**Authors:** Fan Yang, Xinshang Wang, Jingyu Qi, Kun Zhang, Yongli Jiang, Ban Feng, Tao Lv, Le Yang, Qi Yang, Minggao Zhao, Shuibing Liu, Xue Ma

**Affiliations:** 1 Precision Pharmacy & Drug Development Center, Department of Pharmacy, Tangdu Hospital, Fourth Military Medical University, Xi’an 710038, China; 2 Department of Pharmacology, School of Pharmacy, Fourth Military Medical University, Xi’an 710032, China

**Keywords:** depression, diabetes, GLP-1 receptor, microglia, pyroptosis, mitophagy

## Abstract

Depression is a frequent and serious comorbidity associated with diabetes which adversely affects prognosis and quality of life. Glucagon-like peptide-1 receptor (GLP-1R) agonists, widely used in the treatment of diabetes, are reported to exert neuroprotective effects in the central nervous system. Thus, we aim to evaluate whether GLP-1R agonist exendin-4 (EX-4) could alleviate depression-like behaviors in diabetic mice and to explore its underlying mechanism. The antidepressant effects of EX-4 were evaluated using behavioral tests in *db*/*db* mice. The effects of EX-4 on microglial pyroptosis and neuroinflammation were assessed in N9 microglial cells. EX-4 administration alleviated depression-like behaviors in diabetic *db*/*db* mice. GLP-1R activation by EX-4 significantly suppressed microglial pyroptosis and neuroinflammation by downregulation of gasdermin D (GSDMD) and interleukin (IL)-1β in diabetic mice and lipopolysaccharide (LPS)-primed N9 microglia. Mechanistically, GLP-1R activation improved mitochondrial function and promoted mitophagy by decreasing the accumulation of mitochondrial reactive oxygen species (mtROS) and intracellular ROS production. EX-4 exhibits antidepressant effects in depression associated with diabetes in diabetic mice, which may be mediated by inhibiting microglial pyroptisis via promoting mitophagy. It is supposed that GLP-1R agonists may be a promising therapy in depression associated with diabetes.

## 1. Introduction

Depression and diabetes are the leading global causes of morbidity and mortality [1]. One in four patients with type 2 diabetes mellitus (T2DM) has a clinically significant form of depression, and the incidence of depression is twice as high as that in the general population [2]. Similarly, depression is associated with an increased risk of T2DM [3]. Depressive symptoms in T2DM may have adverse effects on diabetes management and glycemic control as well as diabetes-related complications. As a result, the high prevalence of this comorbidity is accompanied by higher rates of mortality worldwide [4]. Although a significant number of patients with diabetes suffer from depression, the majority of them remain untreated [5]. Existing antidepressants and hypoglycemic drugs have been ineffective in alleviating the development of comorbid diabetes and depression, which suggests the urgent need to explore new treatment strategies based on their pathogenic mechanisms.

Chronic inflammation is regarded as the common biological origin contributing to the bidirectional relationship between depression and T2DM [6]. Moreover, neuroinflammation, characterized by the overactivation of glial cells leading to a cytokine-mediated inflammatory response, has been proposed to play an important role in the pathogenesis of depression with diabetes [6,7,8]. Microglia, as the primary immune cells of the central nervous system (CNS), act as the inflammatory signature contributing to neuroinflammation [9]. Microglia-mediated neuroinflammation plays an important role in the progression of neurological diseases and mental disorders. Several studies have revealed that chronic activation of microglia induces the release of proinflammatory cytokines, while overactivation of microglia leads to more severe inflammation-microglial pyroptosis [10,11]. Pyroptosis, a caspase-1-dependent proinflammatory form of programmed cell death, plays vital roles in microglia-mediated neuroinflammation [12]. Activated caspase-1 drives the cleavage of gasdermin D (GSDMD) into *N*-terminal GSDMD domain fragments (GSDMD-N) and the maturation of pro interleukin (IL)-1β into IL-1β. The GSDMD-N domain fragments translocate to the membrane, further bind to the inner membrane lipids to form ring-shaped pores, and lead to the release of mature IL-1β through pores, which triggers pyroptosis and consequential inflammation [12,13]. Meanwhile, recent studies have shown that macrophage pyroptosis is often associated with mitochondrial dysfunction and reactive oxygen species (ROS) generation [14,15]. Current clinical research highlights that the interplay between inflammatory status and mitochondrial function is associated with the severity of depressive symptoms and the complications of diabetes [16,17]. Hence, mitophagy is a promising target for inflammation-related pyroptosis in patients with diabetes and depression.

Glucagon-like peptide-1 (GLP-1) is an endogenous incretin hormone secreted by intestinal cells that activates the GLP-1 receptor (GLP-1R) [18]. In addition to its hypoglycemic effects, GLP-1 and its receptor have many other beneficial actions [19,20]. Exenatide, a GLP-1R agonist, markedly inhibits ROS accumulation and reduces oxidative stress in cardiomyocytes [21]. In CNS, GLP-1R signaling expressed in the microglia was found to be involved in a number of biological processes, including inflammation, neuroprotection, and synaptic plasticity [22]. GLP-1R agonists have been shown to exert neuroprotective effects against microglia-mediated inflammation in neurodegenerative diseases [23,24]. For depression, GLP-1Rs are expressed in neurons of the amygdala and hippocampus, which are known to be depression-associated brain regions [25,26]. However, the role of microglial GLP-1R signaling in the pathophysiology of depressive symptoms and the exact underlying mechanisms have not been fully elucidated.

Given the early findings and pharmacological superiority of GLP-1R agonists in neurological pathophysiology, we hypothesized that GLP-1R activation could inhibit pyroptosis of microglia, subsequently reducing neuroinflammation in diabetes with depression. In the current study, we applied the GLP-1R agonist exendin-4 (EX-4) to verify whether activating GLP-1R could alleviate diabetes with depression by inhibiting the GSDMD-mediated microglial pyroptosis pathway both in vivo and in vitro, thus exploring the underlying mechanism and identifying more efficient therapeutic targets for depression in patients with diabetes.

## 2. Materials and Methods

### 2.1. Reagents and Antibodies

Lipopolysaccharide (LPS, L3023) was purchased from Sigma-Aldrich (St. Louis, MO, USA). Exendin-4 was purchased from APExBIO Technology LLC (Houston, TX, USA). MitoSOX™ Red mitochondrial superoxide indicator (M36008) and BCA Protein Assay Kit (P0914) were purchased from Thermo Fisher Scientific (Waltham, MA, USA). ELISA kits for murine IL-1β (EMC001b.96) were purchased from NeoBioscience (Shenzhen, China). Advanced DMEM medium, fetal bovine serum (FBS), trypsin-EDTA solution, and penicillin-streptomycin solution (100X) were purchased from Gibco (Santa Clara, CA, USA). JC-1 (C2006), ROS (S0033S), and CCK-8 Kit (C0037) were purchased from Beyotime (Shanghai, China).

For Western blot analysis, the following antibodies were used: anti-caspase-1 Ab (1:1000, Proteintech, Wuhan, China, 22915-1-AP); anti-ASC (1:1000, Abcam, Cambridge, UK, ab175449); Ab anti-IL-1β Ab (1:500, Santa Cruz Biotechnology, Dallas, TX, USA, sc-12742); anti-GSDMD Ab (1:1000, Abcam, ab209845); anti-Iba-1 Ab (1:1000, Abcam, ab178846); anti-p62 Ab (1:10,000, Cell Signaling Technology, Boston, MA, USA, 5114); anti-LC3 Ab (1:1000, Cell Signaling Technology, 2775); anti-PINK1 Ab (1:600, Proteintech, 23274-1-AP); anti-Parkin Ab (1:2000, Abcam, ab77924); anti-COX IV Ab (1:1000, Abclonal, Wuhan, China, 23274-1-AP); anti-β-actin Ab (1:10,000, Cell Signaling Technology, 20536-1-AP); goat anti-mouse or rabbit IgG secondary antibody (1:10,000; Thermo, Waltham, MA, USA, 31430 and 31460). 

For immunofluorescence staining analysis, the following antibodies were used: anti-GLP-1R Ab (1:1000, Santa Cruz, sc-390774); anti-Iba-1 Ab (1:1000, Abcam, ab178846); anti-p62 Ab (1:1000, Cell Signaling Technology, 5114); anti-Tomm20 Ab (1:250, Abcam, ab186735); Alexa Fluor 488-conjugated goat anti-rabbit (1:1000, Invitrogen, Carlsbad, CA, USA, A11008); Alexa Fluor 555 goat anti-mouse IgG (1:1000, Invitrogen, 21,422).

### 2.2. Animals 

Male *db*/*db* (BKS.Cg-Dock7m +/+ Leprdb/JNju) and non-diabetic lean control (C57BLKS/JNju) 5-week-old mice (19–35 g) were purchased from Model Animal Research Center of Nanjing University (Nanjing, China). The mice were housed with a 12 h light/dark cycle under controlled temperature (22 ± 2 °C) and humidity (50 ± 10%) and were allowed free access to food and water. All procedures were in accordance with the guidelines approved by the FMMU Animal Care and Use Committee of the Fourth Military Medical University.

### 2.3. Animal Experimental Design

**Figure 1:**
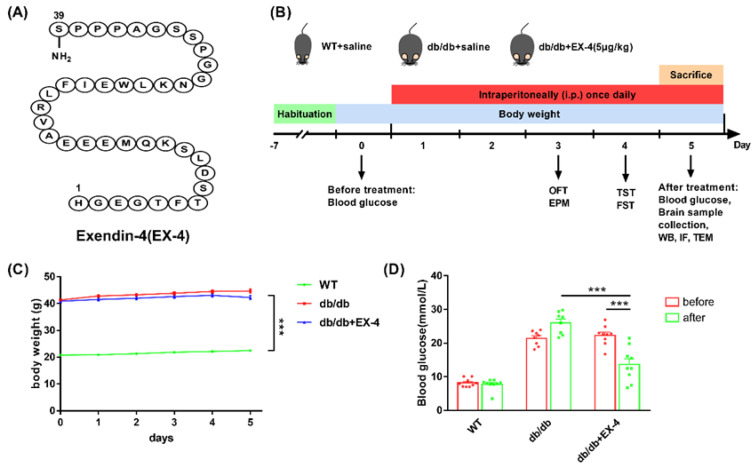
Follow-up with chemical structure of Exendin-4 (EX-4), experimental design, body weight, and blood glucose measurement. (**A**) Chemical structure of EX-4. (**B**) Schematic view of animal experimental design. (**C**,**D**) Body weight and blood glucose levels measured before and after EX-4 treatment. *n* = 8–9 mice/group. Values are shown as the mean ± SEM. *** *p* < 0.001.

Mice were divided randomly into three groups: wild type (WT) + saline, *db*/*db* + saline, *db*/*db* + EX-4; *n* = 9 per group. EX-4 (5 μg/kg) or saline was injected intraperitoneally (i.p.) once daily at regular intervals for four days. Open field test (OFT) and elevated plus maze (EPM) were performed 1 h after EX-4 or saline treatment on day 3. Tail suspension test (TST) and forced swimming test (FST) were performed on day 4 (Figure 1B). The OFT and EPM were used to evaluate anxiety-like behaviors, while the TST and FST were used to assess depression-like behaviors in mice. The behaviors of mice were recorded by a camera and calculated by a video-tracking system (DigBehv-LR4, Shanghai, China). The blood glucose level was determined using a glucometer (SANNUO GA-3, Shenzhen, China).

### 2.4. Behavioral Paradigms

#### 2.4.1. Open Field Test (OFT)

The OFT was performed as previously described [27]. The animals were gently placed in a square arena (30 cm × 30 cm × 30 cm) and were allowed to freely explore for 15 min. 

#### 2.4.2. Elevated plus Maze (EPM)

The EPM test was performed as described in the previous report [27]. The apparatus was composed of two open arms (25 cm × 8 cm × 0.5 cm), two closed arms (25 cm × 8 cm × 12 cm), and a central open platform (8 cm × 8 cm). Mice were placed gently on the central platform, facing an open arm, and allowed to move freely for 5 min. 

#### 2.4.3. Tail Suspension Test (TST)

Animals were suspended by their tails with adhesive tape for 6 min, and the duration of immobility time during the last 4 min was recorded. The duration of immobility time is total time minus struggling time (=immobility time plus the shaking time). 

#### 2.4.4. Forced Swimming Test (FST)

Mice were individually placed in a cylinder (15 cm in diameter) containing 20 cm deep water (23–25 °C) for 6 min. The duration of immobility time in the last 4 min was videotaped.

### 2.5. Cell Culture 

The murine N9 microglial cell line was purchased from Bluefbio (Shanghai, China). The cells were cultured in DMEM containing 10% FBS with penicillin (100 IU/mL) and streptomycin (10 mg/mL) at 37 °C. N9 cells passaged for a maximum of ten times were used in all the experiments. N9 cells were treated with EX-4 for 30 min, and then LPS (1 μg/mL) was added to the supernatant for 24 h.

### 2.6. Cell Viability Assay

CCK-8 assay was performed to evaluate N9 cell viability after treatment. N9 cells were seeded at 5000 in 96-well plates. N9 cells were treated with EX-4 at different concentrations of 1, 10, or 100 nM for 30 min and were then treated with LPS for 24 h. After stimuli, a 10 μL CCK-8 solution was added to each well and incubated for 1 h. Further, the absorbance was measured at 450 nm by a microplate reader.

### 2.7. Western Blot Analysis

Cells and tissue samples were dissociated with sonication in cold RIPA buffer supplemented with phosphatase inhibitor and protease inhibitor. After centrifugation at 4 °C and 12,000 rpm for 20 min, the supernatants were collected and quantified by the BCA Protein Assay Kit. Equal amounts of protein were separated on SDS-PAGE gel and transferred to polyvinylidene fluoride (PVDF) membranes. The membranes were blocked with 5% skimmed milk for 1 h at room temperature and were incubated with primary antibodies overnight at 4 °C. After being washed three times in phosphate-buffered saline-Tween 20 (PBST), the membranes were incubated with horseradish peroxidase-conjugated anti-rabbit or anti-mouse secondary antibodies at room temperature for 1 h and washed in PBST three times. The protein bands were detected by a ChemiDoc XRS (Bio-Rad, Hercules, CA, USA) and were quantified with Image J according to the instructions. The intensity of the bands was analyzed using β-actin as a standard.

### 2.8. Enzyme-Linked Immunosorbent Assay (ELISA)

The concentration of IL-1β was measured by mouse IL-1β ELISA kits according to the manufacturer’s instructions. Supernatant of N9 cells were collected and centrifuged at 2500 rpm for 15 min for analysis. 

### 2.9. Mitochondrial Membrane Potential, Intracellular and Mitochondrial ROS Detection

Mitochondrial membrane potential (Δψm) was assessed by JC-1 staining. The N9 cells were collected in the working solution of JC-1 at 37 °C for 20 min and were washed thrice with the cold-washing buffer. The stained cells were finally analyzed using the Coulter XL (Beckman Coulter, Brea, CA, USA) flow cytometer.

Mitochondrial ROS were measured using MitoSOX™ Red mitochondrial superoxide indicator. The contents (50 μg) were dissolved in 13 μL dimethylsulfoxide (DMSO) to make a 5 mM working solution. After treating the N9 cells with LPS and EX-4, cells were stained with 5 μM MitoSOX™ Red for 10 min at 37 °C. Then, N9 cells were washed with warm PBS and were detected using the Coulter XL (Beckman Coulter, USA) flow cytometer.

After treatment, N9 cells were incubated with 5 μM 2′-7′-dichlorodihydrofluorescein diacetate (CM-DCFHDA) at 37 °C for 20 min. The stained cells were washed twice and were measured using the Coulter XL (Beckman) flow cytometer.

### 2.10. Transmission Electron Microscopy (TEM)

After perfusion, small blocks (∼1 mm^3^) of hippocampal tissue were quickly dissected and post-fixed in the fixation solution at 4 °C for 4 h. The tissues were embedded and cut into ultrathin sections (60–80 nm), which were stained with 2% uranyl acetate and 2.6% lead citrate. The cellular and mitochondrial morphology were examined using a TEM (HT7800, HITACHI, Tokyo, Japan) by a pathologist blinded to the experiments.

### 2.11. Immunofluorescence

The slices were deparaffinized in 3 changes of Biodewax and rehydrated in 3 changes of pure alcohol. The slices were immersed in EDTA antigen retrieval buffer (pH 8.0) to retrieve antigen. After being circled to block endogenous peroxidase, the tissues were blocked with 10% donkey serum at room temperature for 30 min. Then, the slices were placed in a wet box and incubated with the primary antibody overnight at 4 °C. After being covered with secondary antibodies marked with HRP at room temperature for 60 min in dark conditions and being washed with PBS, the slides were incubated with CY3-TSA solution for 10 min, followed by microwave treatment. Then, the slides were incubated with the second primary antibody and second corresponding secondary antibody. The slides were incubated with DAPI solution for 10 min and covered with anti-fade mounting medium. The images were collected by a fluorescent microscope (NIKON Eclipse ci, NIKON, Tokyo, Japan). The morphologies of microglia in the hippocampus were analyzed by the blinded experimenters using software plug-ins AnalyzeSkeleton (2D/3D) in Image J. The endpoints, ramification, and cell shape were summarized in terms of morphology process [28,29].

After stimuli, N9 cells were fixed with 4% paraformaldehyde (PFA) at 4 °C and permeabilized with 0.1% Triton X-100. Cells were incubated with primary antibodies fixed in normal goat serum overnight at 4 °C. After being washed with PBS, N9 cells were incubated with the secondary antibody at room temperature for 1 h. Nuclei were counterstained with DAPI in the dark for 10 min. 

### 2.12. Statistical Analysis

The data were presented as mean ± SEM. The statistical difference was determined by one-way analysis of variance (ANOVA) followed by Tukey’s post hoc test when groups achieved normality and equal variance. If not, Mann–Whitney U tests were used. Correlations and linear regression analysis were used to assess the association between two events. The data were analyzed using GraphPad Prism 7.0. *p* < 0.05 was considered a significant difference.

## 3. Results

### 3.1. Activation of GLP-1R Attenuated Depression-Like Behaviors in db/db Mice

**Figure 2:**
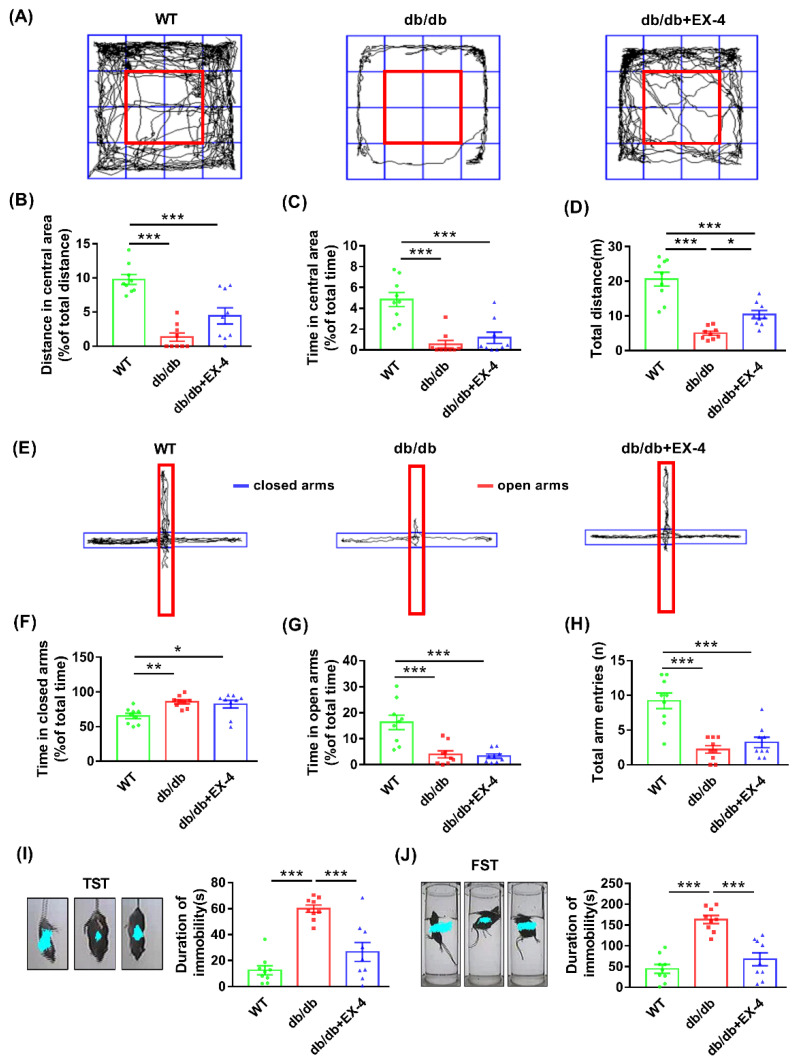
GLP-1R activation ameliorated depression-like behaviors in *db*/*db* mice. (**A**) Representative traces; (**B**,**C**) distance travelled and time spent in the central area; (**D**) total distance in OFT. (**E**) Representative traces; (**F**,**G**) time spent in closed arms and open arms; (**H**) total arm entries in EPM test. The duration of immobility of the observed mice in tail suspension test (TST) (**I**) and forced swimming test (FST) (**J**). *n* = 9 mice/group. Values are shown as the mean ± SEM. * *p* < 0.05, ** *p* < 0.01, *** *p* < 0.001.

To evaluate the effect of GLP-1R on depression-like and anxiety-like behaviors in diabetic mice, age-matched wild-type (WT) mice and *db*/*db* mice were administered saline or the GLP-1R agonist EX-4 at a dose of 5 μg/kg, the chemical structure of which is shown in Figure 1A. The *db*/*db* mice exhibited high blood glucose and body weight; nevertheless, short-term administration of EX-4 only induced effects consistent with the hypoglycemic effect but had no influence on body weight (Figure 1C,D). The OFT showed that *db*/*db* mice displayed decreased total distance travelled, time, and distance in the central area compared to the WT mice (Figure 2A–D). In the EPM test, time spent in the open arms and total arm entries were lower in *db*/*db* mice compared to WT mice. However, EX-4 treatment did not improve anxiety-like behavior in *db*/*db* mice (Figure 2E–H). In the TST and FST, the duration of immobility was significantly prolonged in *db*/*db* mice compared to WT mice. However, this was attenuated in EX-4-treated *db*/*db* mice (Figure 2I,J). These results indicated that GLP-1R activation had an antidepressant effect, but no anxiolytic effect, in *db*/*db* mice.

### 3.2. Microglial Activation in the Hippocampus Was Engaged in the Antidepressant Effects of GLP-1R 

**Figure 3:**
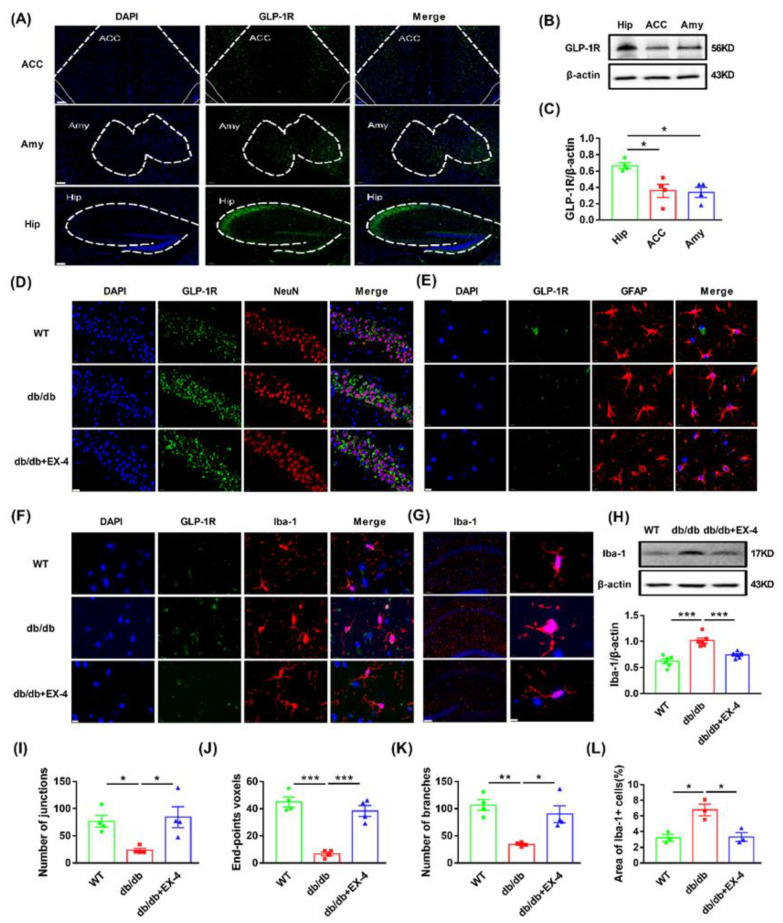
Activation of GLP-1R reduced microglial activation in the hippocampus of *db*/*db* mice. (**A**) Representative micrographs of GLP-1R in the hippocampus, ACC, and amygdala of WT mice. The brain regions were stained with GLP-1R (green) and nuclei were stained with DAPI (blue). Scale bar: 100 μm. (**B**) Representative protein bands of Western blots. (**C**) The protein levels of GLP-1R in the hippocampus, ACC, and amygdala of WT mice. Representative micrographs of GLP-1R (green) in neuron (**D**) (Scale bar: 20 μm), astrocyte (**E**) (Scale bar: 10 μm), and microglia (**F**) (Scale bar: 10 μm) in the hippocampus of WT or *db*/*db* mice after treatment with saline or EX-4. Neuron was stained with NeuN (red), microglia was stained with Iba-1 (red), and astrocyte was stained with GFAP (red). Nuclei were stained with DAPI (blue). (**G**) Representative images of hippocampal microglia in WT or *db*/*db* mice treated with saline or EX-4. Microglia were stained with Iba-1 (red), and nuclei were stained with DAPI (blue). Scale bar (**left**): 100 μm. Scale bar (**right**): 5 μm. (**H**) Representative bands of Western blots and quantification of Iba-1 levels. (**I**–**L**) Morphological analysis of microglia. Area of Iba-1+ cells, number of branches, junctions, and end-points were analyzed by Image J. *n* = 3–5 mice/group. Values are shown as the mean ± SEM. * *p* < 0.05, ** *p* < 0.01, *** *p* < 0.001.

To determine the distribution of GLP-1R in the brain, we first examined the expression of GLP-1R in the hippocampus, amygdala, and anterior cingulate cortex (ACC), which are strongly associated with depression [25,26]. GLP-1R was more highly expressed in the hippocampus than in the amygdala and ACC (Figure 3A–C). To further localize the specific cell type for GLP-1R in the hippocampus, immunofluorescence was stained with cytological markers (anti-NeuN for neurons, anti-Iba-1 for microglia, and anti-GFAP for astrocytes). Although GLP-1R was widely expressed in neurons, microglia, and astrocytes, only microglial GLP-1R was restored by EX-4 in the increased levels of *db*/*db* mice hippocampus. GLP-1R in astrocytes and neurons showed no significant change in *db*/*db* mice compared to EX-4-treated mice (Figure 3D–F). 

Activated microglia plays a vital role in inflammation-mediated depression. They are accompanied by morphological changes, characterized by the retraction of processes and the transformation into amoeba-like shapes [30]. Morphological analysis of tissue slices showed that EX-4 treatment reduced the increased levels of soma size enlargement, thickening, retraction of processes which were represented as the loss of branches, end-point and junctions in hippocampal microglia of *db*/*db* mice (Figure 3G–L). Western blot analysis revealed an increase in Iba-1 expression in the hippocampus of *db*/*db* mice compared to WT mice, which was reversed by EX-4 treatment (Figure 3H). These results demonstrated that microglial activation in the hippocampus might play a major role in the antidepressant effects of GLP-1R.

### 3.3. GLP-1R Activation Suppressed GSDMD-Mediated Pyroptosis in Hippocampal Microglia

**Figure 4:**
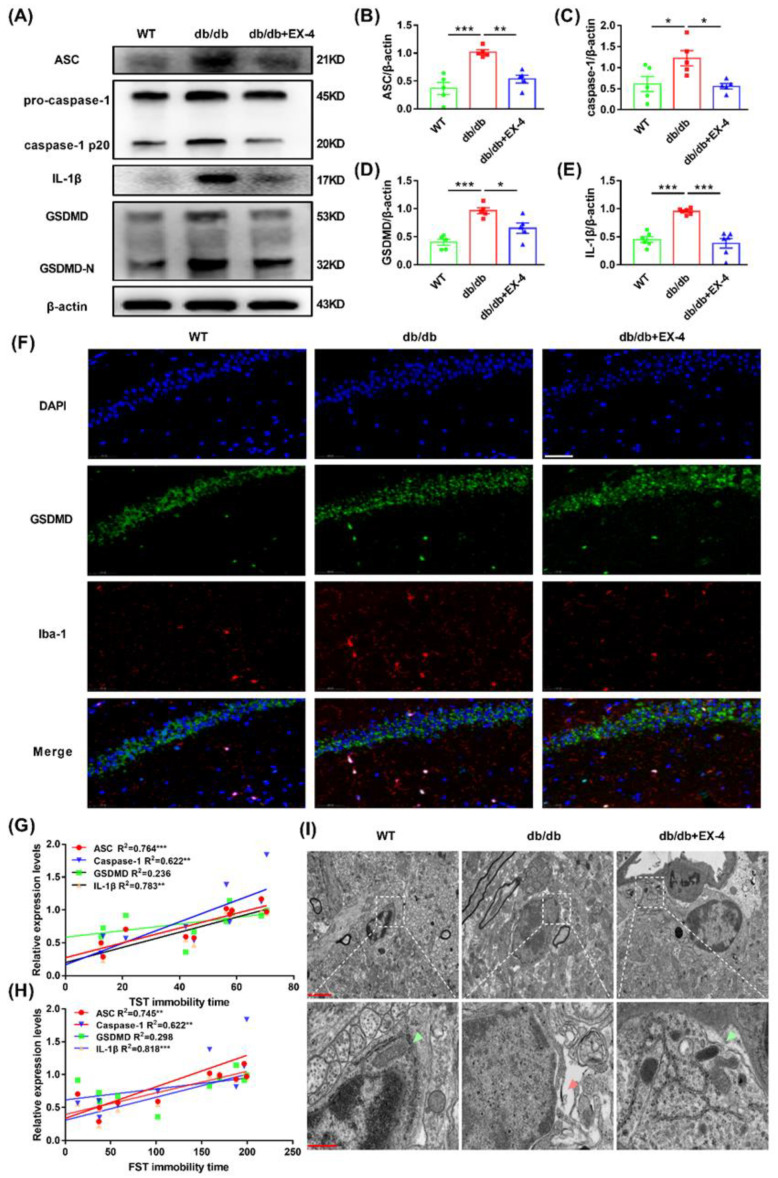
Activation of GLP-1R inhibited GSDMD-mediated microglial pyroptosis in the hippocampus of *db*/*db* mice. (**A**) Representative bands of Western blots. (**B**–**E**) The protein levels of ASC, caspase-1, GSDMD, and IL-1β in the hippocampus of WT or *db*/*db* mice were quantified by Image J. (**F**) Microglia in the hippocampus were stained with GSDMD (green), Iba-1 (red), and DAPI (blue). Scale bar: 50 μm. Correlation between levels of pyroptosis-related proteins and depression-like behaviors TST (**G**) and FST (**H**) in *db*/*db* mice. (**I**) Representative TEM images. The scale bar of upper panels: 2 μm. The scale bar of bottom panels: 500 nm. Green arrow: healthy cellular structure and mitochondria. Red arrow: rupture of cell membrane. *n* = 3–5 mice/group. Values are shown as the mean ± SEM. * *p* < 0.05, ** *p* < 0.01, *** *p* < 0.001.

Excessive activation of microglia triggers pyroptosis, which induces increased release of inflammatory cytokines [15]. To determine the effects of EX-4 on microglial pyroptosis in the hippocampus of *db*/*db* mice, Western blot and immunofluorescence analyses were performed to detect the levels of pyroptosis-related proteins. As shown in Figure 4A–E, the expression of cleaved apoptosis-associated speck-like protein containing CARD (ASC), caspase-1, IL-1β, and GSDMD was dramatically increased in the hippocampus of *db*/*db* mice compared to WT mice, which was reversed by EX-4 treatment. In addition, co-staining of Iba-1 with GSDMD showed that EX-4 inhibited the upregulation of GSDMD in hippocampal microglia of *db*/*db* mice (Figure 4F). We also assessed the correlation between depression-like behavior and pyroposis-related protein levels. There was a positive correlation between immobility time and ASC, caspase-1, and IL-1β levels in both the TST and FST but there was no correlation between immobility time and GSDMD levels (Figure 4G,H). Under TEM, pores of the cell membrane and enlarged perinuclear space in the hippocampal microglia of *db*/*db* mice were observed (Figure 4I).

### 3.4. GLP-1R Activation Suppressed Pyroptosis Induced by LPS in N9 Microglia

**Figure 5:**
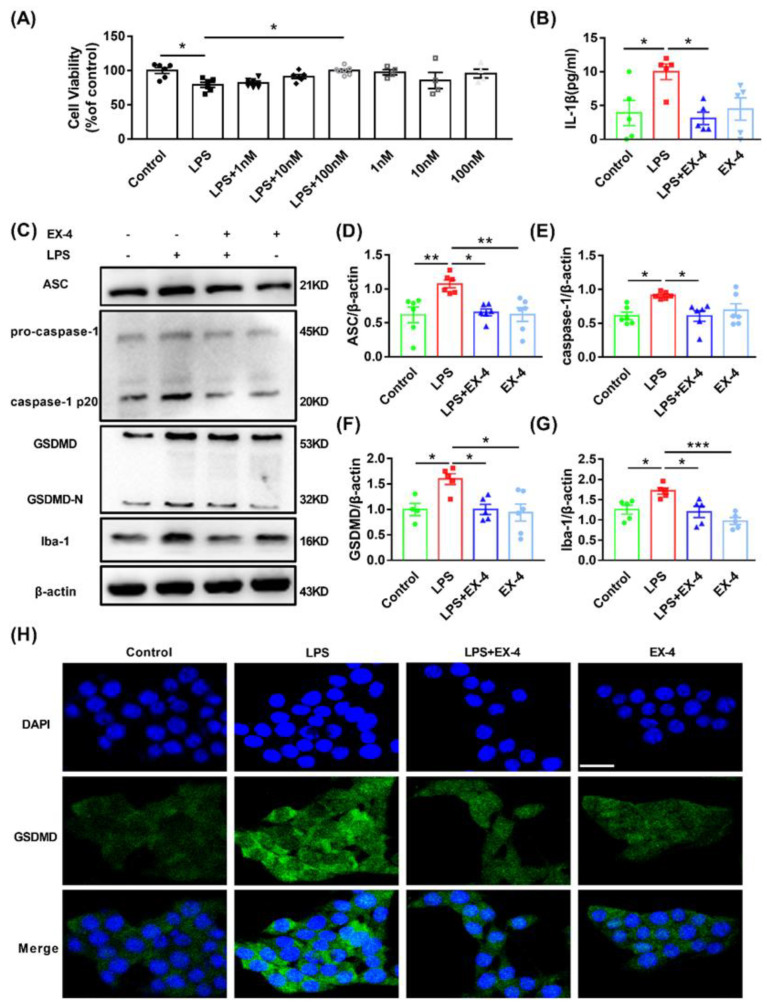
GLP-1R activation inhibited the upregulation of pyroptosis-related protein in LPS-treated N9 microglia. (**A**) N9 cells were treated with different concentrations of EX-4 (1 nm, 10 nm, 100 nm) for 0.5 h and then primed with LPS treatment for 24 h. The 10 μL CCK-8 solution was performed to detect cell viability. (**B**) The levels of IL-1β in supernatants from differently treated N9 cells using ELISA. (**C**) Representative bands of Western blots. (**D**–**G**) The protein expression of ASC, caspase-1, GSDMD, and Iba-1 was determined by Western blot analysis. (**H**) Differently treated N9 cells stained with GSDMD (green) and DAPI (blue) were assessed by immunofluorescence. Scale bar: 20 μm. Values are shown as the mean ± SEM of at least three independent experiments. * *p* < 0.05, ** *p* < 0.01, *** *p* < 0.001.

To confirm the effects of GLP-1R on microglia pyroptosis, N9 cells were pretreated with different concentrations of EX-4 for 0.5 h followed by lipopolysaccharide (LPS) (1 μg/mL) treatment for 24 h. CCK-8 assay showed that EX-4 had a concentration-dependent effect on LPS-treated cytotoxicity. However, only 100 nM EX-4 showed a significant protective effect (Figure 5A). Therefore, we used 100 nM EX-4 to evaluate its effect on microglial pyroptosis in vitro. The release of IL-1β was inhibited by treatment with EX-4, as confirmed by ELISA (Figure 5B). Western blot analysis showed increased levels of ASC, caspase-1, GSDMD, and Iba-1 in N9 cells after LPS treatment, which were all remarkably restored by EX-4 pretreatment (Figure 5C–G). The inhibitory effects of EX-4 on microglial pyroptosis were further verified by immunostaining for GSDMD in vitro (Figure 5H). These results suggested that GLP-1R activation inhibited GSDMD-mediated pyroptosis in N9 microglia cells. 

### 3.5. GLP-1R Activation Ameliorated Mitochondrial and Intracellular ROS Generation by Promoting Mitophagy 

**Figure 6:**
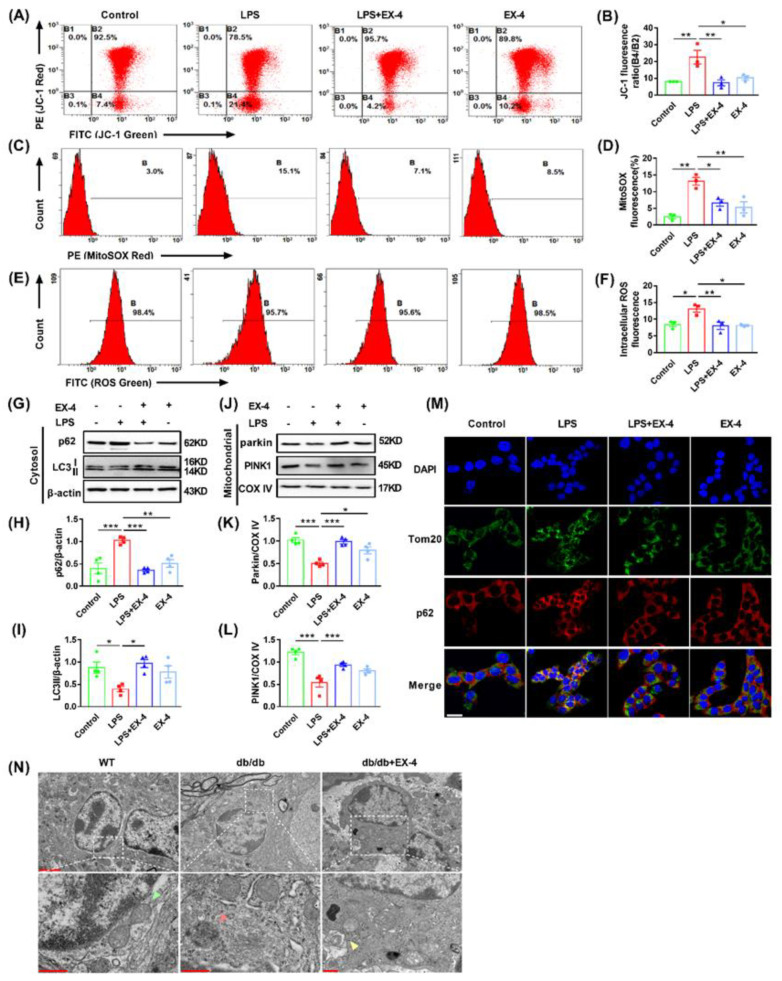
Activation of GLP-1R suppressed mitochondrial and intracellular ROS generation by promoting mitophagy. (**A**) Scatter diagram of JC-1 staining in N9 cells using flow cytometer. (**B**) The quantitation of mitochondrial membrane potential in differently treated N9 cells (B4/B2 ratio). (**C**,**D**) Scatter diagram of MitoSOX™ Red mitochondrial superoxide indicator was detected through flow cytometer and quantitative to the ratio of mitochondrial ROS (mtROS). (**E**,**F**) DCFH-DA staining was performed to analyze the quantitation of intracellular ROS fluorescence intensity using flow cytometer. (**G**–**L**) Representative bands of Western blot (**G**,**J**), quantitative analysis of p62 and LC3II in the cytosol (**H**,**I**), and the analysis of parkin and PINK1 in the mitochondrial (**K**,**L**). (**M**) Representative immunofluorescence images of p62 (red), Tom20 (green), and DAPI (blue) in differently treated N9 microglia cells. Scale bar: 20 μm. (**N**) TEM images of the hippocampal microglia of WT or *db*/*db* mice treated with saline or EX-4. Green arrow: healthy mitochondria. Red arrow: damaged mitochondria. Yellow arrow: mitophagosome. The scale bar of upper panels: 2 μm. The scale bar of bottom panels: 500 nm. Data are shown as the mean ± SEM of at least three independent experiments. * *p* < 0.05, ** *p* < 0.01, *** *p* < 0.001.

Damaged mitochondria are the main source of ROS, and excessive ROS production promotes the production of more inflammatory cytokines and exacerbates inflammation and inflammation-associated pyroptosis. To determine whether EX-4 was responsible for ROS production and mitochondrial dysfunction, LPS-treated N9 microglia were analyzed using a flow cytometer. JC-1 staining showed that EX-4 pretreatment preserved an impaired Δψm as a decrease in the green JC-1 monomeric form in the R4 region (Figure 6A,B). Furthermore, LPS induced increased ratio of mitochondrial ROS production and intensity of intracellular ROS in microglia through MitoSOX^™^ and DCFH-DA assays, while the accumulation of intracellular and mitochondrial ROS was inhibited by treatment with EX-4 (Figure 6C–F). The above data showed that activating GLP-1R suppressed mitochondrial dysfunction, thereby ameliorating ROS production, which aggravated microglial pyroptosis and enhanced the release of inflammatory cytokines.

Dysfunctional mitochondria are eliminated by mitophagy to maintain the mitochondrial function. As a type of selective autophagy, mitophagy is controlled by PTEN-induced kinase 1 (PINK1), parkin, and other quality control proteases for the timely removal of damaged or superfluous mitochondria. To investigate whether mitophagy participates in the regulation of EX-4 in ROS production and microglial pyroptosis, the expression of autophagic and mitophagy-related protein markers was evaluated in LPS-primed N9 cells. Immunoblot analysis showed that in the cytosol, the level of microtubule-associated protein light chain 3 (LC3) II decreased, whereas the expression of p62 increased significantly in the LPS-treated group. Treatment with EX-4 markedly reversed these effects (Figure 6G–I). The loss of PINK1 and Parkin in the mitochondria by LPS alone was also reversed by EX-4 pretreatment (Figure 6J–L). Furthermore, we examined mitophagosomes by co-immunostaining for p62 and Tomm20, a protein that labels the outer membrane of mitochondria. Consistent with the Western blot analysis, co-localization of p62 with Tomm20 increased in LPS-treated cells, which was suppressed by EX-4 pretreatment (Figure 6M). Likewise, TEM showed mitochondrial swelling, decrease or disappearance of mitochondrial crista, and vacuolization in the hippocampal microglia of *db*/*db* mice, which were partially ameliorated by EX-4 treatment. The mitophagosome was also found in the hippocampal microglia of *db*/*db* mice treated with EX-4 (Figure 6N). Taken together, these findings indicated that EX-4 promoted mitophagy in microglia, which reduced damaged mitochondria and microglial pyroptosis both in vivo and in vitro.

## 4. Discussion

**Figure 7:**
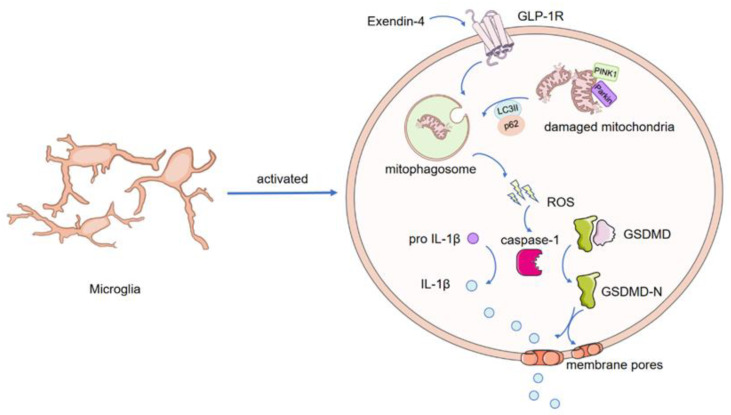
A graphical abstract of the molecular mechanisms underlying the antidepressant effect of GLP-1R.

In the present study, we demonstrated for the first time that activation of GLP-1R significantly ameliorated depression-like behaviors in diabetic *db*/*db* mice by inhibiting microglial pyroptosis and promoting mitophagy in the hippocampus. We showed that suppression of mitochondrial dysfunction and promotion of mitophagy are the pathways by which GLP-1R decreases ROS accumulation for final pyroptosis activation (Figure 7). Our study focused on alleviating microglia-mediated neuroinflammation in depression, which might help in the clinical application of GLP-1R agonists in the treatment of depression associated with diabetes.

Depression has become a common comorbidity in people with diabetes, and the prevalence of diabetes with depression is substantially increasing [2]. However, existing therapeutic agents are not effective in alleviating the development of diabetes and depression comorbidity. Although there have been several findings suggesting that chronic inflammation is a common link between diabetes and depression [7,31], few studies have attempted anti-inflammation approaches for the treatment of diabetes with depression [32].

GLP-1R agonists are now widely used in the treatment of patients with T2DM. In addition, the activation of GLP-1R has anti-inflammatory effects in several organs, suggesting that it may be also useful in the treatment of inflammatory diseases [33]. There is growing evidence that GLP-1R activation exerts neuroprotective effects on oxidative stress, neuronal apoptosis, and neuroinflammation in patients with Alzheimer’s disease (AD) or Parkinson’s disease (PD) [34]. Several clinical evidences have demonstrated that GLP-1R agonists can improve cognitive function in T2DM patients through anti-inflammatory effects [34]. Of concern, neuroinflammation may be strongly associated with diabetes,, while exacerbating mood disorders including depression [35]. Although few studies have shown that GLP-1R agonists may be valuable in the treatment of depression, the exact causal relationship and mechanism of the antidepressant and anti-diabetic effects of GLP-1 agonists have not been elucidated [36]. 

Here, we evaluated the role of the GLP-1R agonist in brains of *db*/*db* mice, an established animal model of type 2 diabetes associated with mood disorders [37]. Treatment with GLP-1R agonist EX-4 markedly ameliorated the depression-like behavior in *db*/*db* mice, suggesting that GLP-1R is involved in the depression modulation in diabetic mice. Increasing evidence indicates that the inflammatory response is a main factor involved in the development of depression with diabetes [38,39]. Previous studies have found that hippocampal inflammation in *db*/*db* mice is associated with increased anxiety-like behavior [40]. Microglia in several brain regions, especially the hippocampus, might be linked to the inflammatory response in diabetic depression [41]. Furthermore, the change of hippocampal volume is associated with the duration of depressive symptoms and the use of antidepressants [42]. In this study, GLP-1R was expressed in all these three depression-related regions but only highly expressed in the hippocampus, suggesting GLP-1R in the hippocampus may play a crucial role. This is consistent with previous studies showing that the hippocampus is the key structure responsible for the regulation and control of emotion [43]. We further found the area of GFAP+ and NeuN+ cells had no change in each group, while the area of Iba-1+ cells had significant differences in *db*/*db* mice and EX-4 treated mice. It suggested that microglial GLP-1R signaling-mediated neuroinflammation may be closely related to the development of diabetes with depression. To conclude, we focused on the change of microglia in the hippocampus of *db*/*db* mice for the further study of depression in vivo.

Excessive or persistent activation of microglia leads to neuroinflammation in the hippocampus, which may be associated with PD, AD, depression, and other CNS diseases [44,45]. In our morphologic study, the decrease in branch number, branch length, and end-point voxels via 2D/3D skeleton analysis showed microglia activation in the hippocampus of *db*/*db* mice. Interestingly, we found that activating GLP-1R suppressed the activation of microglia in vivo as well as LPS-treated N9 microglia in vitro. Pro caspase-1 binds to apoptosis-associated speck-like protein containing CARD (ASC) via CARD–CARD interaction and subsequently cleaves GSDMD into GSDMD *N*-terminal domain to form plasma membrane pores. They can also cleave inactive pro IL-1β into mature IL-1β, releasing pro-inflammatory cytokines into the extracellular environment, which amplificate the inflammatory response [12,13]. Excessive activation of microglia aggravates neuroinflammation and subsequently leads to microglial pyroptosis, which is associated with multiple CNS diseases [46,47]. LPS-induced and chronic social defeat stress (CSDS) depression models have also reported the link with elevated caspase-1 and GSDMD [48]. Herein, we found the increased level of caspase-1, ASC, GSDMD, and IL-1β in the hippocampus of *db*/*db* mice as well as in LPS-treated N9 cells. The rupture of the microglial membrane was also observed in TEM images, indicating that EX-4 treatment dramatically inhibited microglial pyroptosis. To further confirm the effect of EX-4 and locate in microglial GLP-1R, co-immunostaining of Iba-1 with GSDMD in the hippocampus of *db*/*db* mice was applied and showed the same results. Thus, we determined that microglial pyroptosis-related neuroinflammation may be the therapeutic target for the effect of GLP-1R on diabetes with depression.

Mitochondria are considered the main source of ROS in the cell [49]. The current clinical study suggested that the interaction between inflammatory status and mitochondrial function is associated with the severity of depressive symptoms [16]. Mitochondrial dysfunction and the accompanying accumulation of ROS have been gradually used as one of the biomarkers of the pathophysiology process of major depressive disorder [16]. Increased levels of mitochondrial fission genes were observed in streptozotocin (STZ)-induced diabetic mice with depression [50]. In our experiment, activation of microglial GLP-1R inhibited mitochondrial dysfunction and thus ameliorated ROS production. The balance between ROS production and ROS scavenging relies on the cellular clearance and degradation pathway, called autophagy [51]. Mitophagy is a selective autophagy, which eliminates damaged mitochondria in mitophagosomes and ultimately controls mitochondrial quality [52]. Elimination of mitophagy has been indicated that it resulted in damaged mitochondria signals, such as the accumulation of mitoROS and damaged mitochondria, thus leading to pyroptosis and the release of IL-1β, which contributes to the progression of neurodegenerative diseases [53,54]. However, mitophagy, a promising targeted strategy, has not been focused on the inflammation of diabetes with depression. In our present study, we determined that EX-4 had a robust effect against the change of LC3B and p62, both in mitochondria and in cytoplasm, suggesting the role of GLP-1R on the impairment of the bulk autophagy. PINK1 and Parkin, as mitophagy-related protein, not only respond to the loss of mitochondrial membrane potential via the clearance of damaged mitochondria, but also participate in the regulation of apoptotic cascade (including mitochondria Bax translocation and apoptosis-related protein caspase 9 and caspase 3) [55,56]. Our results showed that LPS-treated N9 microglia indicated lower expression of PINK1 and Parkin, which is consistent with recent study of patients with major depressive disorder. Interestingly, we found that EX-4 upregulated PINK1 and Parkin levels, which inhibited the activation of caspase-1-dependent pyroptosis, and that it is associated with inflammatory response. The remarkable finding is that we observed mature mitophagosome and the process of mitochondria fusing with lysosome in the hippocampus of *db*/*db* mice via TEM, suggesting mitophagy is supposed to mediate the effect of GLP-1R on caspase-1-dependent microglia pyroptosis and accompanying inflammation.

## 5. Conclusions

Our findings support previous studies on the role of microglial pyroptosis in depression psychopathology in order to identify their importance in the antidepressant effect of activation of GLP-1R. The data indicated that activation of GLP-1R alleviated depression-like behaviors and associated neuroinflammation through inhibiting GSDMD-mediated microglial pyroptosis by promoting mitophagy in the depressive hippocampus. This study might provide new therapeutic targets for depression with diabetes.

## Data Availability

The data that support the findings of this study are available from the corresponding author upon reasonable request.
